# The clinical relevance of plasma potassium abnormalities on admission in trauma patients: a retrospective observational study

**DOI:** 10.1186/s40560-015-0103-6

**Published:** 2015-08-13

**Authors:** Takaaki Ookuma, Koji Miyasho, Nobuhiro Kashitani, Nobuhiko Beika, Naoki Ishibashi, Takahiro Yamashita, Yoshihito Ujike

**Affiliations:** Fukuyama City Hospital Emergency Medical Center, 5-23-1 Zaocho, Fukuyama, Hiroshima 721-8511 Japan; Department of Emergency and Critical Care Medicine, Field of Functional Physiology, Branch of Biophysiological Science, Okayama University Graduate School of Medicine, Dentistry, and Pharmaceutical Science, Okayama, 700-8558 Japan

**Keywords:** Trauma, Potassium, Hyperkalemia, Hypokalemia, Mortality, Intervention, Craniotomy

## Abstract

**Background:**

Abnormalities in potassium levels can lead to several clinical difficulties in trauma patients admitted to the ICU. However, the significance of potassium abnormalities soon after admission in trauma patients has not yet been clearly delineated. The objective of this study was to describe the plasma potassium abnormalities in trauma patients on admission and to examine the clinical outcomes associated with these abnormalities.

**Methods:**

We performed a retrospective observational study of plasma potassium levels in trauma patients admitted to the Fukuyama City Hospital between January 1, 2010 and December 31, 2013. Five hundred twenty consecutive trauma patients were included and categorized into six groups according to their plasma potassium level on admission (<3.0, 3.0–<3.5, 3.5–<4.0, 4.0–<4.5, 4.5–<5.0, and ≥5.0 mEq/L). After adjusting for covariates, including age, gender, the Revised Trauma Score, and the Injury Severity Score, logistic regression analysis was used to examine the association between plasma potassium levels and outcomes, including life-saving interventions and in-hospital mortality.

**Results:**

Two hundred twenty-seven patients (43.7 %) presented with hypokalemia (<3.5 mEq/L), while seven patients (1.3 %) presented with hyperkalemia (≥5.0 mEq/L). Patients in the lowest potassium group (<3.0 mEq/L, *n* = 36 [6.9 %]) were significantly associated with craniotomy (adjusted odds ratio 5.25 [95 % confidence interval 2.06–13.40]; *p* < 0.001) and showed an increased trend toward in-hospital mortality. In the second lowest potassium group (3.0–< 3.5 mEq/L, *n* = 191 [36.7 %]), the adjusted odds ratio for craniotomy was significantly higher (2.03 [95 % confidence interval 1.01–4.07]; *p* = 0.048) compared to the reference group.

**Conclusions:**

Trauma patients presenting with hypokalemia (<3.5 mEq/L) on admission may be associated with severe head trauma requiring life-saving craniotomy.

## Background

Various studies about potassium abnormalities in trauma patients have been performed to assess its significance and to elucidate its mechanism [1–4]. Hyperkalemia is recognized as a general phenomenon in trauma patients because of the theory that the release of cellular contents due to the tissue damage or hemorrhagic shock commonly occurs in the severely injured patients [5, 6]. On the other hand, hypokalemia has been reported as a more general and significant phenomenon and has been investigated for its clinical importance and basic mechanism [7–12]. The main mechanism of post-traumatic hypokalemia is considered that potassium shifts into the cells responding to epinephrine surge caused by injury.

Although several studies have tried to elucidate the association between the potassium abnormalities and outcomes in trauma patients, the significance of the potassium abnormalities soon after admission in trauma patients has not been clearly delineated.

We hypothesize that the occurrence of potassium abnormalities in trauma patients soon after admission is associated with the outcomes of these patients. We herein describe the plasma potassium abnormalities in trauma patients on admission and examine the clinical outcomes associated with these abnormalities in a retrospective observational study.

## Methods

### Study design

This study was a retrospective observational study conducted at the Fukuyama City Hospital. The Fukuyama City Hospital is a community hospital with 506 beds, located in the eastern area of Hiroshima prefecture in Japan, with an Emergency Medical Center corresponding to the tertiary trauma center in this area. This study was approved by the Institutional Review Board of the Fukuyama City Hospital.

### Patients

All trauma patients with neither cardio-pulmonary arrest on arrival nor burn injury were eligible if they had been transported to the Fukuyama City Hospital between January 1, 2010 and December 31, 2013 and if their Injury Severity Score (ISS) was greater than 15 [13].

The patients were excluded if they met the following criteria: (1) were transported to the Fukuyama City Hospital after 24 h or later from the injury onset, (2) the dates when they were injured were unknown, (3) the serum creatinine values on admission were greater than 2 mg/dL, or (4) a blood gas analysis was not performed on admission.

### Demographic and clinical information

Information was obtained from the hospital medical records and our database of trauma patients conforming to the Japan Trauma Data Bank and included the following: age, sex, type of trauma, situation of transport, vital signs on admission, laboratory values, injury profile according to the Abbreviated Injury Score (AIS), ISS, and therapeutic interventions, including surgery [14, 15].

### Scoring for trauma severity

To assess the severity of trauma, the following scores were coded and calculated by trained assistants and physicians. To assess the anatomical severity, AIS was coded by the AIS90 update98 and ISS was calculated [15]. To assess the physiologic severity, the Revised Trauma Score (RTS) was calculated according to the vital signs on admission [16]. Using the method of the Trauma Score and the Injury Severity Score (TRISS), we estimated the probability of survival to describe the study population [17].

### Measurement of potassium and other values

In the trauma medicine department at the Fukuyama City Hospital, blood sampling is typically performed on admission for the analysis of blood gas, laboratory tests and quick preparation for blood transfusions. The analysis of blood gas was performed with ABL700 (Radiometer, Copenhagen, Denmark), and we obtained the following values of the plasma from this analysis: potassium, sodium, pH and bicarbonate. The serum creatinine level was measured in the Hospital Central Laboratory Division with LABOSPECT 008 (Hitachi High-Technologies Corporation, Tokyo, Japan).

### Outcome

The primary outcome was death during the hospitalization from all causes. The secondary outcome was the occurrence of a life-saving intervention (LSI). According to the current literature available on the epidemiology of trauma deaths, we categorized the LSI into 2 groups, the LSI for neurological emergency and the LSI for bleeding [18, 19].

The LSI for neurological emergency was defined as craniotomy. The LSI for bleeding was defined as intra-aortic balloon occlusion, transcatheter arterial embolization, massive transfusion, thoracotomy or laparotomy. The transcatheter arterial embolization and surgeries in the LSI were defined as those performed within 24 h of arrival. The intra-aortic balloon occlusion was defined as that performed between the arrival and start of hospitalization. A massive transfusion was defined as a transfusion of 10 or more units of packed red blood cells within 24 h of arrival.

### Statistical analysis

Continuous variables were assessed for graphically normality and are described as the mean (standard deviation) or median [inter-quartile range]. Categorical variables are described as n (%). To adjust for the severity of trauma, the RTS was divided into two groups (<4 and ≥4) and the ISS was divided into three groups (16–24, 25–40, and 41–75) [16].

The normal range of potassium was set between 3.5 and <5.0 mEq/L for describing the distribution of the potassium disturbance. Additionally, six groups of different potassium levels by 0.5 mEq/L was created to investigate the association of potassium concentration with the clinical characteristics and outcomes (<3.0, 3.0–<3.5, 3.5–<4.0, 4.0–<4.5, 4.5–<5.0, and ≥5.0 mEq/L).

We used the following tests to compare the characteristics between the six potassium groups: Fisher’s exact test for categorical variables, the Kruskal-Wallis test for continuous variables without normality, and a one-way analysis of variance for continuous variables with normality. We used a logistic regression analysis to examine the association between the potassium levels and outcomes while adjusting for covariates, including age, gender, the RTS, and the ISS. In this logistic regression analysis, the potassium concentration of 3.5–<4.0 mEq/L was set at the reference group. The results of the logistic regression analyses were reported using the odds ratio (OR) and 95 % confidence interval (CI).

All tests were two sided, and statistical significance was considered to exist at *p* < 0.05. All analyses were performed using the R software package (version 3.1.1).

## Results

**Fig. 1:**
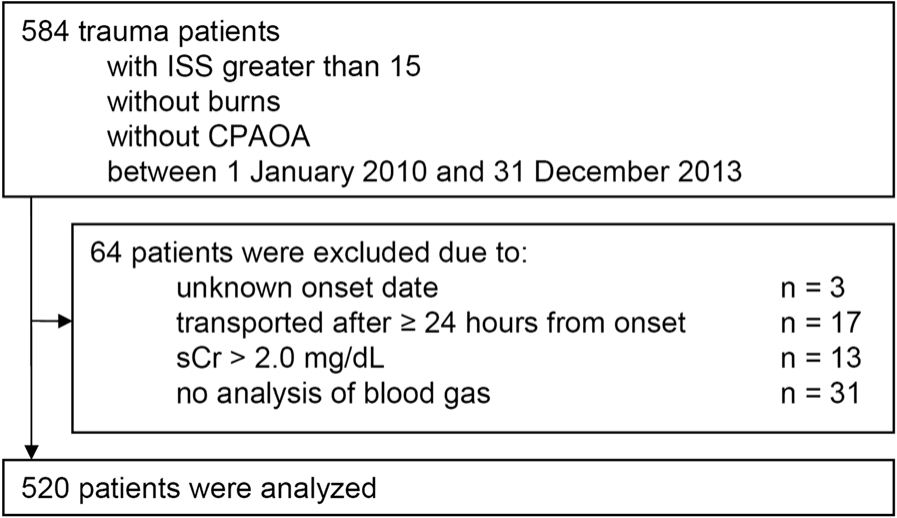
Flow chart of the patient selection. *ISS* Injury Severity Score, *CPAOA* cardio-pulmonary arrest on arrival, *sCr* serum creatinine

Between January 1, 2010 and December 31, 2013, 584 of all trauma patients transported to the Fukuyama City Hospital had an ISS of more than 15 and were neither cardio-pulmonary arrest on arrival nor burn patients (Fig. 1). Of these patients, 64 were excluded, 17 were transported to the hospital after 24 h or later from injury, 3 had no information when they were injured, 13 had serum creatinine values greater than 2 mg/dL on admission, and 31 did not have a blood gas analysis on admission. Ultimately, 520 patients were analyzed.

Table 1 shows the characteristics of the study population. The range of the potassium concentration on admission was 2.49–5.72 mEq/L, and 227 patients (43.7 %) presented with hypokalemia (<3.5 mEq/L), while 7 patients (1.3 %) presented with hyperkalemia (≥5.0 mEq/L). The median of the ISS was 25, and the median of the RTS was 7.84. The median of the probability of survival calculated by the TRISS method was 0.89. During hospitalization, 44 patients (8.5 %) died.

**Table 1:** Characteristics of the study population

Characteristics	Value
No. of patients	520
Age, year, median [IQR]	61 [35, 74]
Sex, male, *n* (%)	355 (68.3)
Type of trauma	
Blunt injury, *n* (%)	510 (98.1)
Penetrating injury, *n* (%)	10 (1.9)
Transferred from another hospital, *n* (%)	111 (21.3)
Laboratory values upon admission	
Potassium, mEq/L, mean [range]	3.61 [2.49, 5.72]
Hypokalemia (<3.5 mEq/L), *n* (%)	227 (43.7)
Normokalemia (3.5–<5.0 mEq/L), *n* (%)	286 (55.0)
Hyperkalemia (≥5.0 mEq/L), *n* (%)	7 (1.3)
Scores	
ISS, median [IQR]	25 [19, 30]
RTS, median [IQR]	7.84 [6.49, 7.84]
TRISS Ps, median [IQR]	0.89 [0.73, 0.95]
LSI for bleeding^a^, *n* (%)	117 (22.5)
Craniotomy, *n* (%)	64 (12.3)
In-hospital mortality, *n* (%)	44 (8.5)

*IQR* inter-quartile range, *ISS* Injury Severity Score, *RTS* Revised Trauma Score, *TRISS Ps* probability of survival calculated by the method of the Trauma Score and the Injury Severity Score, *LSI* life-saving intervention

^a^The LSI for bleeding was defined as intra-aortic balloon occlusion, transcatheter arterial embolization, massive transfusion, thoracotomy, or laparotomy

We divided the patients into six groups according to the potassium levels on admission (Table 2). In the groups with extreme potassium levels (<3.0 and ≥5.0 mEq/L), the Glasgow Coma Scale and the RTS tended to be lower. The LSI for bleeding tended to be conducted more frequently in the higher potassium groups. Craniotomy tended to be conducted frequently in the lowest potassium group (12/36, 33.3 %). The gross in-hospital mortality was highest in the highest potassium group (2/7, 28.6 %) and lowest in the group with a potassium level of 3.0–<3.5 mEq/L (8/191, 4.2 %).

**Table 2:** Characteristics and outcomes by the potassium level on admission

Potassium level on admission (mEq/L)	<3.0	3.0–<3.5	3.5–<4.0	4.0–<4.5	4.5–<5.0	≥5.0	*p* value
No. of patients (%)	36 (6.9)	191 (36.7)	195 (37.5)	78 (15)	13 (2.5)	7 (1.3)	
Age, year, median [IQR]	38 [16, 61.75]	53 [28.5, 69.5]	64 [42, 74.5]	68.5 [60.5, 80]	75 [56, 86]	51 [44, 70]	<0.001
Sex, male, *n* (%)	26 (72.2)	119 (62.3)	137 (70.3)	61 (78.2)	10 (76.9)	2 (28.6)	0.03
Transferred from another hospital, *n* (%)	6 (16.7)	30 (15.7)	49 (25.1)	20 (25.6)	4 (30.8)	2 (28.6)	0.14
Vital signs on admission							
SBP, median [IQR]	139 [119.25, 157.5]	131 [109, 148]	134 [110.5, 156]	136 [104.75, 155]	107 [77, 119]	122 [92.5, 123.5]	0.04
Heart rate, median [IQR]	94 [78.5, 111.5]	90 [75.5, 105]	86 [73.5, 101]	89.5 [73.5, 106.5]	96 [81, 115]	101 [80, 111]	0.45
RR, median [IQR]	22 [15.75, 30]	20 [16, 24]	20 [15, 25]	20.5 [16.25, 27.75]	23 [18, 30]	14 [14, 19.5]	0.27
GCS, median [IQR]	8.5 [3, 13]	15 [11, 15]	15 [13, 15]	15 [13, 15]	14 [7, 15]	5 [3, 11]	<0.001
Temperature, °C, median [IQR]	36.05 [35.65, 36.52]	36.20 [35.50, 36.70]	36.20 [35.50, 36.80]	36.20 [35.62, 36.60]	35.60 [35.50, 36.60]	35.90 [35.25, 36.90]	0.79
Scores							
ISS, median [IQR]	26 [21.75, 41.5]	25 [19, 29]	25 [19, 30]	26 [21, 34]	25 [20, 34]	25 [21.5, 27.5]	0.39
RTS, median [IQR]	5.97 [4.09, 7.55]	7.84 [6.61, 7.84]	7.84 [6.90, 7.84]	7.84 [6.69, 7.84]	7.11 [5.23, 7.55]	5.03 [3.73, 6.90]	<0.001
TRISS Ps, median [IQR]	0.80 [0.39, 0.94]	0.91 [0.78, 0.97]	0.89 [0.78, 0.94]	0.86 [0.66, 0.93]	0.86 [0.33, 0.89]	0.63 [0.42, 0.87]	<0.001
Outcomes							
LSI for bleeding^a^, *n* (%)	7 (19.4)	33 (17.3)	47 (24.1)	21 (26.9)	5 (38.5)	4 (57.1)	0.05
Craniotomy, *n* (%)	12 (33.3)	25 (13.1)	16 (8.2)	7 (9.0)	3 (23.1)	1 (14.3)	<0.01
In-hospital mortality, *n* (%)	7 (19.4)	8 (4.2)	14 (7.2)	10 (12.8)	3 (23.1)	2 (28.6)	<0.01

*IQR* inter-quartile range, *SD* standard deviation, *SBP* systemic blood pressure, *GCS* Glasgow Coma Scale, *ISS* Injury Severity Score, *RR* respiratory rate, *RTS* Revised Trauma Score, *TRISS Ps* probability of survival calculated by the method of the Trauma Score and the Injury Severity Score, *LSI* life-saving intervention

^a^The LSI for bleeding was defined as intra-aortic balloon occlusion, transcatheter arterial embolization, massive transfusion, thoracotomy or laparotomy

The logistic regression analysis was performed to examine the association between the plasma potassium levels and outcomes, with the potassium level of 3.5–<4.0 mEq/L set as the reference group (Table 3). The unadjusted ORs for the mortality and craniotomy were significantly higher in the lowest potassium group (<3.0 mEq/L) (OR for mortality 3.12 [95 % CI 1.16–8.38]; *p* = 0.024, OR for craniotomy 5.59 [95 % CI 2.36–13.20]; *p* < 0.001). After adjusting for covariates, the patients in the lowest potassium group (<3.0 mEq/L) maintained a significantly higher OR for craniotomy (5.25 [95 % CI 2.06–13.40]; *p* < 0.001), while no group showed statistical significance about in-hospital mortality. In the second lowest potassium group (3.0–<3.5 mEq/L), the adjusted OR for craniotomy was significantly higher (2.03 [95 % CI 1.01–4.07]; *p* = 0.048) compared to the reference group. The odds ratios for the LSI for bleeding tended to be higher in the higher potassium groups, while no statistical significance was detected in the adjusted ORs.

**Table 3:** Logistic regression for the outcomes

Potassium level on admission (mEq/L)	<3.0		*p* value		3.0–<3.5		*p* value	3.5–<4.0	4.0–<4.5	*p* value	4.5–<5.0	*p* value	≥5.0	*p* value
Unadjusted OR (95 % CI)														
LSI for bleeding		0.76 (0.31–1.85)		0.545		0.66 (0.40–1.08)	0.099	1 [ref]	1.16 (0.64–2.11)	0.627	1.97 (0.61–6.31)	0.254	4.20 (0.91–19.40)	0.067
Craniotomy		5.59 (2.36–13.20)		<0.001		1.68 (0.87–3.27)	0.123	1 [ref]	1.10 (0.44–2.79)	0.836	3.36 (0.84–13.40)	0.087	1.86 (0.21–16.50)	0.575
In-hospital mortality		3.12 (1.16–8.38)		0.024		0.57 (0.23–1.38)	0.210	1 [ref]	1.90 (0.81–4.48)	0.142	3.88 (0.96–15.70)	0.058	5.17 (0.92–29.10)	0.062
Adjusted OR (95 % CI)														
LSI for bleeding		0.48 (0.18–1.29)		0.145		0.69 (0.41–1.18)	0.178	1 [ref]	1.05 (0.55–2.00)	0.893	1.45 (0.36–5.82)	0.604	2.79 (0.54–14.40)	0.220
Craniotomy		5.25 (2.06–13.40)		<0.001		2.03 (1.01–4.07)	0.048	1 [ref]	1.02 (0.39–2.66)	0.974	2.93 (0.62–13.90)	0.176	1.36 (0.14–13.00)	0.788
In-hospital mortality		3.03 (0.89–10.30)		0.077		0.79 (0.30–2.09)	0.630	1 [ref]	1.57 (0.60–4.14)	0.359	1.20 (0.17–8.79)	0.854	3.17 (0.40–25.20)	0.276

*OR* odds ratio, *CI* confidence interval, *LSI* life-saving intervention, *ref* reference

## Discussion

In this retrospective observational study, we found that the patients presenting with hypokalemia (<3.5 mEq/L) had significantly more incidence of craniotomy as a surrogate of the severity of head trauma.

Several studies investigated the clinical significance of post-traumatic hypokalemia regarding the response of the sympathetic nervous system to head trauma and suggested that a certain degree of the response indicates a poorer outcome [8, 9, 12]. Beal et al. reported that hypokalemia was common in trauma patients and associated with some features including younger age, head trauma, lower Glasgow Coma Scale (GCS), higher ISS, and others [10]. They also categorized the severity of hypokalemia and found that patients with severe hypokalemia (<3.1 mEq/L) had lower admission GCS than those who had mild hypokalemia (3.4–3.5 mEq/L). In the present study, we categorized the study cohorts by the potassium levels on admission and found that the lowest potassium group (<3.0 mEq/L) had the highest OR for craniotomy with a statistical significance, while the second lowest potassium group (3.0–<3.5 mEq/L) also had the adjusted OR for craniotomy with a statistical significance. Unfortunately, we were not able to consider the association between the detailed information about head injury requiring craniotomy and the severity of hypokalemia in the present study. However, our results suggest that the severity of post-traumatic hypokalemia may reflect the severity of head injury and that a certain degree of post-traumatic hypokalemia has the possibility to be an indicator of severe head trauma requiring life-saving craniotomy.

The mechanism of post-traumatic hypokalemia has been gradually elucidated; however, whether the post-traumatic hypokalemia resolves without any intervention remains unknown. Epinephrine most likely induced hypokalemia because potassium normally shifts into the cells responding to epinephrine [20]. Beal et al. reported that the epinephrine levels on admission were higher in trauma patients with hypokalemia [11]. They also found that the epinephrine levels decreased toward the normal level simultaneously with the recovery of hypokalemia after 24–36 h. However, Wu et al. reported in the observational study of the hypokalemic patients with traumatic brain injury that hypokalemia mainly occurred later than the first 24 h after injury, and considerable numbers of patients presented with severe hypokalemia [12]. In the present study, we observed 6.9 % of all trauma patients presented with severe hypokalemia (<3.0 mEq /L) and the lowest value of potassium of 2.49 mEq/L, although the information about the courses of the potassium abnormalities was unfortunately unavailable. If the post-traumatic hypokalemia was caused by the potassium shifts and the reaction ceased shortly, the recovery of hypokalemia may be expected without any intervention. However, in the cases of prolonged or considerable hypokalemia, some of the interventions for hypokalemia may be required. Although the other various causes are possible, the prolonged post-traumatic hypokalemia itself is possible to reflect the prolonged derangement of neurological condition caused by head trauma.

We observed no significant association between clinical outcome and hyperkalemia soon after admission. However, we found that the patients with higher potassium levels tended to require interventions for bleeding. Post-traumatic hyperkalemia is induced by extensive tissue damage and aggressive transfusion [6]. Additionally, prolonged hemorrhagic shock theoretically leads to hyperkalemia due to alterations in the cellular membrane function [5]. The main causes of death due to trauma include injury of the central nervous system and exsanguination; the latter is related to the extensive tissue damage and hemorrhagic shock [18, 19]. In the present study, most of the patients were injured by a blunt mechanism related to the extensive tissue damage and many patients had brain injury; hence, the two main pathologies mentioned above may coexist. Therefore, the effect of hyperkalemia may be offset by the effect of hypokalemia, and the clinical significance of hyperkalemia may be obscured.

There are several limitations associated with this study. First, we collected the potassium concentration from the analysis of blood gas in the emergency room, which was a measurement of the plasma. The potassium concentration in the serum may result in a higher value than in the plasma, which is caused by the potassium release from platelets or other cells [21]. Therefore, care should be taken when the results of the present study are extrapolated to other investigations or compared with other studies. Contrary, the internal validity of the present study is presumed to be unchanged, because we chose the measurement of the plasma in which the dispersion of potassium values from this phenomenon was consequently assumed to be smaller. Second, this was a retrospective observational study, and thus, it is possible that some of the important factors that may affect the results were overlooked. These factors include prescribed medicines, kidney function, pH, interval from injury to admission, infusion or transfusion before admission, and interventions for potassium abnormalities before admission. The detailed medical information such as the medicines or the interventions before admission was unfortunately unavailable in this study because of the paucity and uncertainty of information stemmed from the retrospective design. We tried to minimize the effects of them by setting and adjusting the design of this study. For example, the kidney function was considered by the creatinine level to exclude the patients with renal insufficiencies possible to affect the metabolism of potassium, and the temporal or medical concerns were considered by setting the time window of inclusion. Third, a small sample size made the statistical analysis difficult, especially in the groups with extreme potassium levels, and may have decreased the power of analysis. Further investigation with considerations to the points mentioned above is required to evaluate the role of potassium abnormalities as informative and valuable phenomena in trauma medicine.

## Conclusions

In conclusion, we herein found that trauma patients presenting with hypokalemia (<3.5 mEq/L) required increased incidence of craniotomy and hypokalemia (<3.0 mEq/L) showed an increased trend toward in-hospital mortality in a retrospective observational study. Our findings suggest that hypokalemia in trauma patients on admission reflects the severity of head trauma.

## Abbreviations

AIS: Abbreviated Injury Score
CI: confidence interval
ISS: Injury Severity Score
LSI: life-saving intervention
OR: odds ratio
RTS: Revised Trauma Score
TRISS: Trauma Score and the Injury Severity Score

## References

[1] Desborough JP (2000). The stress response to trauma and surgery. Br J Anaesth.

[2] Zavagli G, Pampolini M, Cavallini G, Cavallesco G, Ricci G (1988). Different kalemia in abdominal trauma. J Trauma.

[3] Morell V, Lundgren E, Gillott A (1993). Predicting severity of trauma by admission white blood cell count, serum potassium level, and arterial pH. South Med J.

[4] Vanek VW, Seballos RM, Chong D, Bourguet CC (1994). Serum potassium concentrations in trauma patients. South Med J.

[5] Illner HP, Cunningham JN, Shires GT (1982). Red blood cell sodium content and permeability changes in hemorrhagic shock. Am J Surg.

[6] Perkins RM, Aboudara MC, Abbott KC, Holcomb JB (2007). Resuscitative hyperkalemia in noncrush trauma: a prospective, observational study. Clin J Am Soc Nephrol.

[7] Shin B, Mackenzie CF, Helrich M (1986). Hypokalemia in trauma patients. Anesthesiology.

[8] Pomeranz S, Constantini S, Rappaport ZH (1989). Hypokalaemia in severe head trauma. Acta Neurochir (Wien).

[9] Lazar L, Erez I, Gutermacher M, Katz S (1997). Brain concussion produces transient hypokalemia in children. J Pediatr Surg.

[10] Beal AL, Scheltema KE, Beilman GJ, Deuser WE (2002). Hypokalemia following trauma. Shock.

[11] Beal AL, Deuser WE, Beilman GJ (2007). A role for epinephrine in post-traumatic hypokalemia. Shock.

[12] Wu X, Lu X, Lu X, Yu J, Sun Y, Du Z (2015). Prevalence of severe hypokalaemia in patients with traumatic brain injury. Injury.

[13] Baker SP, O'Neill B, Haddon W, Long WB (1974). The injury severity score: a method for describing patients with multiple injuries and evaluating emergency care. J Trauma.

[14] Koseki K, Mashiko K, Sakamoto T, Miyake Y, Saitoh D, Fujita H (2004). Activity of trauma registry committee and future perspectives. J Jpn Assoc Surg Trauma.

[15] Japanese Association for the Surgery of Trauma, Japan Automobile Research Institute (2003). AIS90 Update 98 Japanese translation.

[16] Champion HR, Sacco WJ, Copes WS, Gann DS, Gennarelli TA, Flanagan ME (1989). A revision of the Trauma Score. J Trauma.

[17] Boyd CR, Tolson MA, Copes WS (1987). Evaluating trauma care: the TRISS method. Trauma Score and the Injury Severity Score. J Trauma.

[18] Baker CC, Oppenheimer L, Stephens B, Lewis FR, Trunkey DD (1980). Epidemiology of trauma deaths. Am J Surg.

[19] Evans JA, van Wessem KJ, McDougall D, Lee KA, Lyons T, Balogh ZJ (2010). Epidemiology of traumatic deaths: comprehensive population-based assessment. World J Surg.

[20] Reid JL, Whyte KF, Struthers AD (1986). Epinephrine-induced hypokalemia: the role of beta adrenoceptors. Am J Cardiol.

[21] Nijsten MW, de Smet BJ, Dofferhoff AS (1991). Pseudohyperkalemia and platelet counts. N Engl J Med.

